# Temperature-Dependent Dynamic Characteristics of Carbon-Fiber-Reinforced Plastic for Different Spectral Loading Patterns

**DOI:** 10.3390/ma13225238

**Published:** 2020-11-19

**Authors:** Chan-Jung Kim

**Affiliations:** Department of Mechanical Design Engineering, Pukyong National University, 45 Yongso-ro, Nam-gu, Busan 48513, Korea; cjkim@pknu.ac.kr

**Keywords:** carbon-fiber-reinforced plastic material, modal damping coefficient, resonance frequency, temperature condition, direction of carbon fiber

## Abstract

The dynamic properties of carbon-fiber-reinforced plastic (CFRP) can be efficiently estimated through a modal damping coefficient and a resonance frequency, and the modal parameters can be calculated using a frequency response function (FRF). The modal parameters used in an CFRP FRF are influenced by the carbon fiber direction, temperature, and spectral loading pattern, as well as the operating conditions. In this study, three parameters—temperature, spectral loading pattern, and carbon fiber direction—were selected as the influential factors for CFRP dynamics, and the sensitivity index formulation was derived from the parameter-dependent FRF of the CFRP structure. The derivatives of the parameter-dependent FRF over the three considered parameters were calculated from the measured modal parameters, and the dynamic sensitivity of the CFRP specimens was explored from the sensitivity index results for five different directional CFRP specimens. The acceleration response of a simple CFRP specimen was obtained via a uniaxial excitation test at temperatures ranging from −8 to 105 °C for the following two spectral loading cases: harmonic and random.

## 1. Introduction

Carbon-fiber-reinforced plastic (CFRP) can be used as an excellent lightweight replacement for steel in mechanical industries [1,2,3,4,5] due to its outstanding specific strength characteristics [6,7,8,9,10,11], as well as its sound damping characteristics [12,13,14,15,16,17]. The dynamic response of a CFRP structure varies with the changes in structural stiffness with respect to the direction of the carbon fiber; hence, CFRP is anisotropic in nature due to the different orientations of the carbon fiber [18,19,20,21,22,23,24]. In addition, there are several weaving processes for producing CFRP products, such as plain-weave, twill-weave, and unidirectional-weave, to create a three-dimensional structure; sometimes, a combination of different weaving methods is utilized [20,21,22,23,24]. Therefore, compared to isotropic materials such as steel, the industrial utilization of CFRP is challenging due to its anisotropic property arising from the weaving method and the direction of the carbon fiber.

The mechanical components, mass (or inertia), damper and spring, are required to express the physical quantities of the linear mechanical structure so that the damping and spring coefficients can be determined as equivalent values according to the degree of freedom of the linear model. The damping and spring coefficients are typically identified via an experimental process or can be estimated as theoretical parameters via a system modeling process. The two responsible coefficients can be measured in the time or frequency domains, and the measurement method is selected depending on which domain the dynamic response is required to be in. To obtain the spring coefficient in the time domain, it is generally possible to measure the amplitude of displacement as the input load increases, as well as to estimate it within the scheduled load range. In the case of a damping coefficient, the attenuation value of the response during one period of the oscillating signal can be used to obtain the damping coefficient in the time domain, and the measured value is only valid for the applied single frequency [25,26,27]. Measuring the damping coefficient in the time domain requires considerable time and cost; such methods are applied in limited cases where precise damping coefficients are required by taking the nonlinear properties into account [25,26,27]. When the dynamic response is evaluated in the frequency domain, the modeling process can be simplified under the assumption that the nonlinear properties are negligible. In the frequency domain, it is generally possible to use the FRF to identify the system dynamics by estimating both the system parameters—the damping and spring coefficients—from the enlarged curve for each resonance frequency. The FRF can be simply measured by using an impact hammer or exciter, and all the damping and spring coefficients can be simultaneously identified from the measured FRF. Therefore, in this study, the modal damping coefficient was used to measure the damping property of CFRP under the assumption of a linear system instead of taking measurements in a time domain.

CFRP exhibits anisotropic properties over the direction of carbon fiber, and the modal parameters of CFRP are varied with respect to several parameters. In some previous studies [22,28,29], sensitivity to the FRF was assessed according to different carbon fiber directions and specific loading patterns to evaluate the dynamic properties of CFRP. In another previous study, a simple specimen test was performed on different carbon fiber directions, and the results of the test were evaluated to determine the sensitivity of temperature conditions to the CFRP [24]. Previous studies have revealed that the dynamics of CFRP specimens are highly sensitive to the three parameters of interest: temperature, loading pattern, and carbon fiber direction. However, the analytical results revealed that the dynamics of CFRP specimens were sensitive to only two parameters: temperature and carbon fiber direction. In the present study, the investigation of the dynamics of CFRP was extended to consider all three parameters—temperature, loading pattern, and carbon fiber direction—and the variations in modal parameters, i.e., the resonance frequency and damping coefficient, were evaluated using the sensitivity index of the FRF of the CFRP specimen. A uniaxial vibration test was conducted for the simple CFRP specimen under two spectral loading patterns, harmonic and random, and the frequency range was set to contain at least one resonance frequency of the responsible specimen. The FRFs were calculated using the measured data and modal parameters of the CFRP specimen. Both the resonance frequency and the damping coefficient were estimated from these FRFs.

## 2. Estimation Method for the Modal Damping Coefficient

In a linear system, the FRF between the input signal, and the response is used to efficiently represent the dynamic characteristics of the linear system. For linear systems with a single input and output, the ratio of the response (*R*(*ω*)) to the externally held input (*F*(*ω*)) is expressed as follows, under the *ω* (Hz) condition for the *i*th mode:

(1) $$\frac{R\left(\omega \right)}{F\left(\omega \right)}=\frac{R_{i}^{e}}{-M_{i}\omega^{2}+C_{i}\omega j+K_{i}}$$

where $R_{i}^{e}$, $M_{i}$, $C_{i}$, and $K_{i}$ represent the residual, equivalent mass, damping coefficient, and stiffness coefficient in the *i*th mode, respectively. The resonance frequency in the *i*th mode can be defined as $\omega_{n,i}\left(=\sqrt{K_{i}/M_{i}}\right)$, and the modal damping coefficient can be written as follows [25,26,27]:

(2) $$\xi_{i}=\frac{C_{i}}{2\sqrt{M_{i}K_{i}}}$$

The modal damping coefficient $\xi_{i}$ can also be experimentally obtained using the two half-power points represented as $\omega_{n,i}^{\left(1\right)}$ and $\omega_{n,i}^{\left(2\right)}$, and it can be presented as follows [25,26,27]:

(3) $$\xi_{i}=\frac{\left|\omega_{n,i}^{\left(2\right)}-\omega_{n,i}^{\left(1\right)}\right|}{\omega_{n,i}}$$

The FRF (*H*(*ω*)) can be modified using the modal parameters, i.e., the resonance frequency and modal damping coefficient, as follows:

(4) $$H\left(\omega \right)=\frac{r_{i}^{e}}{{\left(\omega_{n,i}\right)}^{2}-\omega^{2}+2\omega_{n,i}\omega \xi_{i}j}$$

where $r_{i}^{e}$ represents the normalized residual. According to a previous study [22], the modal parameters, the modal damping coefficient $\xi_{i}$, and resonance frequency $\omega_{n,i}$, in the FRF in CFRP specimen were dependent on the direction of the carbon fiber and temperature, respectively. Hence, it was verified that $\xi_{i}$ was dependent on both the spectral loading pattern and the direction of the carbon fiber; however, $\omega_{n,i}$ was only dependent on the carbon fiber direction. Thus, the FRF of linear systems for one input and output in all the frequency bands of interest is expressed as follows, based on the summation of modes theory:

(5) $$H\left(\omega ,\theta ,T,p\right)=\overset{N}{\underset{i=1}{\sum }}\frac{r_{i}^{e}}{{\left(\omega_{n,i}\left(\theta ,T\right)\right)}^{2}-\omega^{2}+2\omega_{n,i}\left(\theta ,T\right)\omega \xi_{i}\left(\theta ,T,p\right)j}$$

Sensitivity analysis is an efficient method to identify the influence of certain parameters on the target system or objective function; hence, the minimum design modification can be attained using sensitivity analysis results [30,31,32,33,34,35,36]. The dynamic sensitivity of CFRP can be evaluated over the three parameters of interest using the partial derivative of the parameter-dependent FRF in Equation (5). The sensitivity analysis formulation was derived as the partial derivative of the FRF over three parameters involved in the dynamic characteristics of the system: these are the carbon fiber angle (*θ*), temperature (*T*), and spectral loading pattern (*p*), which can be expressed, respectively, as follows:

(6) $$\begin{array}{l} \frac{\partial H\left(\omega ,\theta ,T,p\right)}{\partial \theta }\\ =\overset{N}{\underset{i=1}{\sum }}\frac{2r_{i}^{e}}{{\left[{\left(\omega_{n,i}\left(\theta ,T\right)\right)}^{2}-\omega^{2}+2\omega_{n,i}\left(\theta ,T\right)\omega \xi_{i}\left(\theta ,T,p\right)j\right]}^{2}}\;\{\omega_{n,i}\left(\theta ,T\right)\frac{\partial \omega_{n,i}\left(\theta ,T\right)}{\partial \theta }\\ +\left(\omega \xi_{i}\left(\theta ,T,p\right)\frac{\partial \omega_{n,i}\left(\theta ,T\right)}{\partial \theta }+\omega_{n,i}\left(\theta ,T\right)\omega \frac{\partial \xi_{i}\left(\theta ,T,p\right)}{\partial \theta })j\right\}\; \end{array}$$

(7) $$\begin{array}{l} \frac{\partial H\left(\omega ,\theta ,T,p\right)}{\partial T}\\ =\overset{N}{\underset{i=1}{\sum }}\frac{2r_{i}^{e}}{{\left[{\left(\omega_{n,i}\left(\theta ,T\right)\right)}^{2}-\omega^{2}+2\omega_{n,i}\left(\theta ,T\right)\omega \xi_{i}\left(\theta ,T,p\right)j\right]}^{2}}\;\{\omega_{n,i}\left(\theta ,T\right)\frac{\partial \omega_{n,i}\left(\theta ,T\right)}{\partial T}\\ +\left(\omega \xi_{i}\left(\theta ,T,p\right)\frac{\partial \omega_{n,i}\left(\theta ,T\right)}{\partial T}+\omega_{n,i}\left(\theta ,T\right)\omega \frac{\partial \xi_{i}\left(\theta ,T,p\right)}{\partial T}\right)j\} \end{array}$$

(8) $$\frac{\partial H\left(\omega ,\theta ,T,p\right)}{\partial p}\;=\overset{N}{\underset{i=1}{\sum }}\frac{2r_{i}^{e}}{{\left[{\left(\omega_{n,i}\left(\theta ,T\right)\right)}^{2}-\omega^{2}+2\omega_{n,i}\left(\theta ,T\right)\omega \xi_{i}\left(\theta ,T,p\right)j\right]}^{2}}\;\left\{\omega_{n,i}\left(\theta ,T\right)\omega \frac{\partial \xi_{i}\left(\theta ,T,p\right)}{\partial p}j\right\}$$

The sensitivity index in the *i*th mode can be derived from the previous formulation by only considering the variation terms for each parameter, which can be defined, respectively, as follows [22]:

(9) $$I_{\theta ,i,k}=\left|\omega_{n,i}\left(\theta_{k},T\right)\frac{\partial \omega_{n,i}\left(\theta_{k},T\right)}{\partial \theta }+\left(\omega \xi_{i}\left(\theta_{k},T,p\right)\frac{\partial \omega_{n,i}\left(\theta_{k},T\right)}{\partial \theta }+\omega_{n,i}\left(\theta_{k},T\right)\omega \frac{\partial \xi_{i}\left(\theta_{k},T,p\right)}{\partial \theta }\right)j\right|$$

(10) $$I_{T,i,k}=\left|\omega_{n,i}\left(\theta ,T_{k}\right)\frac{\partial \omega_{n,i}\left(\theta ,T_{k}\right)}{\partial T}+\left(\omega \xi_{i}\left(\theta ,T_{k},p\right)\frac{\partial \omega_{n,i}\left(\theta ,T_{k}\right)}{\partial T}+\omega_{n,i}\left(\theta ,T_{k}\right)\omega \frac{\partial \xi_{i}\left(\theta ,T_{k},p\right)}{\partial T}\right)j\right|$$

(11) $$I_{p,i,k}=\left|\omega_{n,i}\left(\theta ,T\right)\omega \frac{\partial \xi_{i}\left(\theta ,T,p_{k}\right)}{\partial p}j\right|$$

where *k* represents the *k*-th case. Finally, the sensitivity analysis for the CFRP specimens could be efficiently analyzed via the normalized sensitivity index of each specimen over the summation of all possible cases. From the previous indices for each parameter of interest, the normalized sensitivity index can be expressed, respectively, as:

(12) $${\bar{I}}_{\theta ,i}=\frac{I_{\theta ,i,k}}{\sum_{k}I_{\theta ,i,k}}$$

(13) $${\bar{I}}_{T,i}=\frac{I_{T,i,k}}{\sum_{k}I_{T,i,k}}$$

(14) $${\bar{I}}_{p,i}=\frac{I_{p,i,k}}{\sum_{k}I_{p,i,k}}$$

## 3. Uniaxial Excitation Test

**Figure 1:**
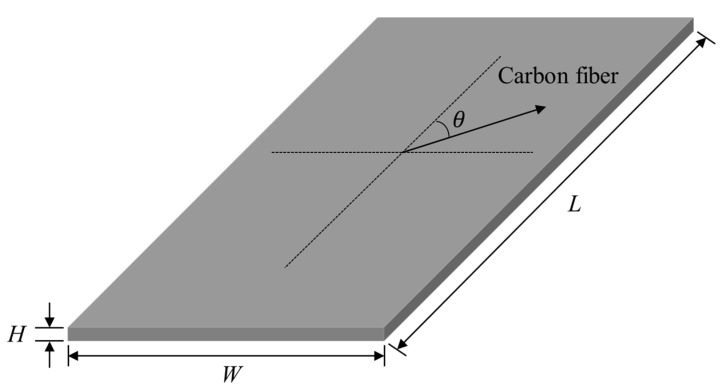
Configuration of the simple rectangular carbon-fiber-reinforced plastic (CFRP) specimen: The dimensions of the designed specimen were 80 mm (*W*), 150 mm (*L*), and 3 mm (*H*).

A simple rectangular specimen (80 mm × 150 mm × 3 mm) was prepared to evaluate the dynamic sensitivity over the three parameters of interest, as shown in Figure 1. To minimize the effect of dynamics from the external shape, the configuration of the CFRP specimen was designed to be simple, and the following five carbon fiber directions were selected: 0°, 30°, 45°, 60°, and 90°. Pre-implemented composite fibers were produced by SK Chemical (USN 250A, Seongnam, South Korea) using carbon fibers produced by Toray (T700(12k), Tokyo, Japan) and by using epoxy resin as the binding polymer. The stacking sequence was unidirectional, and the composite specimen (thickness: 3 mm) was fabricated with 12 layers of the pre-implemented composite fibers (thickness: 0.258 mm), in which the weight fractions of the carbon fibers and the resin were 64% and 36%, respectively. By cutting the large base material according to the five desired directions of carbon fiber, the experimental error that may have occurred due to different characteristics of the base material was minimized.

Uniaxial vibration tests were performed using a uniaxial exciter (LW127.141-225, Labworks, Costa Mesa, CA, USA) to observe the dynamic behavior of the CFRP specimens. Two spectral loading patterns—random and harmonic—were applied for the uniaxial excitation test, and a maximum frequency of 500 Hz was selected, accounting for the resonance of the CFRP specimen. Table 1 and Table 2 present the profiles that were used in the uniaxial excitation test and temperature condition was summarized in Table 3.

**Figure 2:**
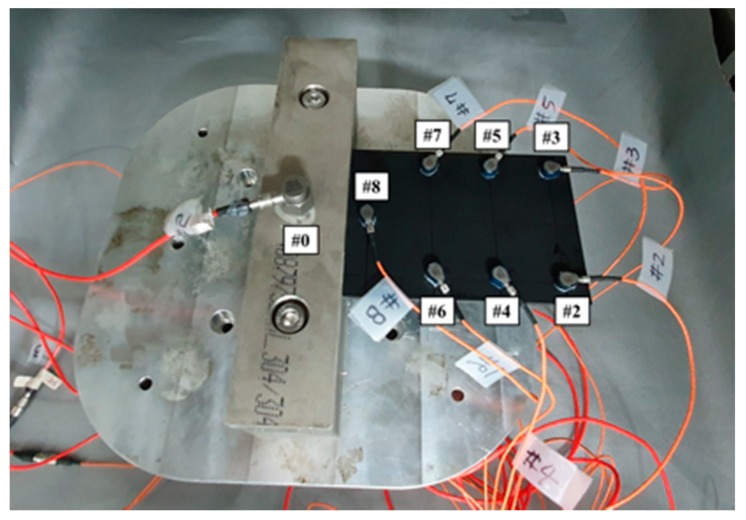
Location of the acceleration sensors: Seven locations (#2–#8) were selected to measure the dynamics of the CFRP specimen, and one location (#0) was selected for the exciter controller.

**Figure 3:**
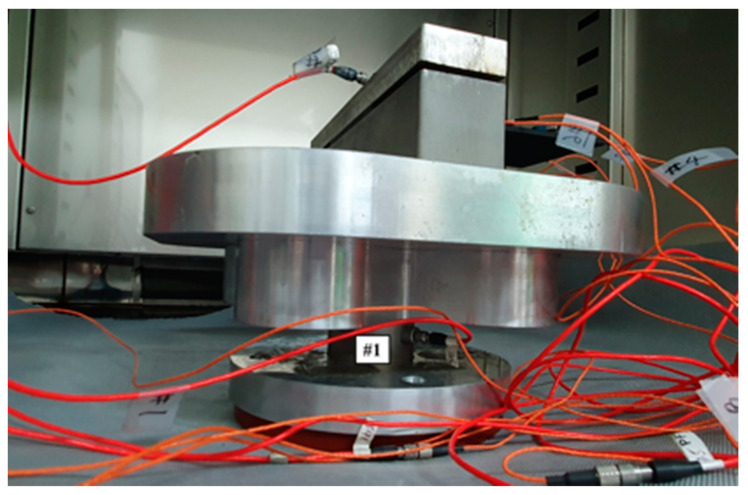
Location of the force sensor: the force sensor was attached beneath the clamping jig.

**Figure 4:**
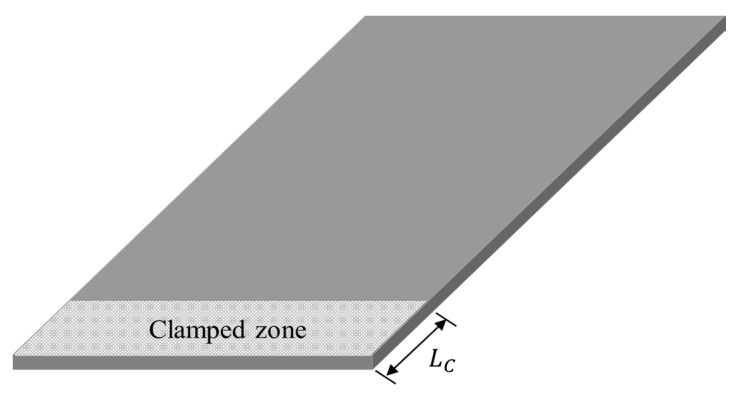
Configuration of the clamped zone in the CFRP specimen: *L*_C_ = 40 mm.

The tests were conducted for five different carbon fiber direction conditions (*θ*_1_ = 0°, *θ*_2_ = 30°, *θ*_3_ = 45°, *θ*_4_ = 60°, and *θ*_5_ = 90°), two excitation cases (*p*_1_ = harmonic and *p*_2_ = random) and five temperature conditions (*T*_1_ = −8 °C, *T*_2_ = 20 °C, *T*_3_ = 50 °C, *T*_4_ = 80 °C, and *T*_5_ = 105 °C). The measurement was simultaneously performed for both the input load (#1) and the response acceleration (#2–#8) at different locations to measure the FRF, as shown in Figure 2 and Figure 3. The temperature parameter was controlled using an environmental chamber (Model: EN-VTH-602-V, 9.5 kW capacity, ENEX SCIENCE, Goyang, South Korea) integrated in a uniaxial exciter, and the effective inner dimension of the environmental chamber was 600 mm × 600 mm × 600 mm. The data acquisition equipment was Test. Lab (Siemens, Munich, Germany), and the load and acceleration sensors were 1061V1 (Dytran, Chatsworth, CA, USA) and 3225F2 (Dytran, Chatsworth, CA, USA), respectively. The acceleration sensors collected measurements at seven different locations; thus, seven FRFs could be measured for each service test. The acceleration sensors were sufficiently spaced to account for the characteristics of the CFRP specimen, and the acceleration data on channel #0 were attached separately to control the uniaxial exciter. The CFRP specimen jig, fabricated from SUS304 material, was firmly fixed at a depth of 40 mm on one side of the specimen, as illustrated in Figure 4. The fixed boundary condition at the end of the CFRP specimen was selected to locate its first resonance frequency within the excitation frequency range (from 10 to 500 Hz) [24] while safely transmitting the excitation energy through the clamped jig.

**Figure 5:**
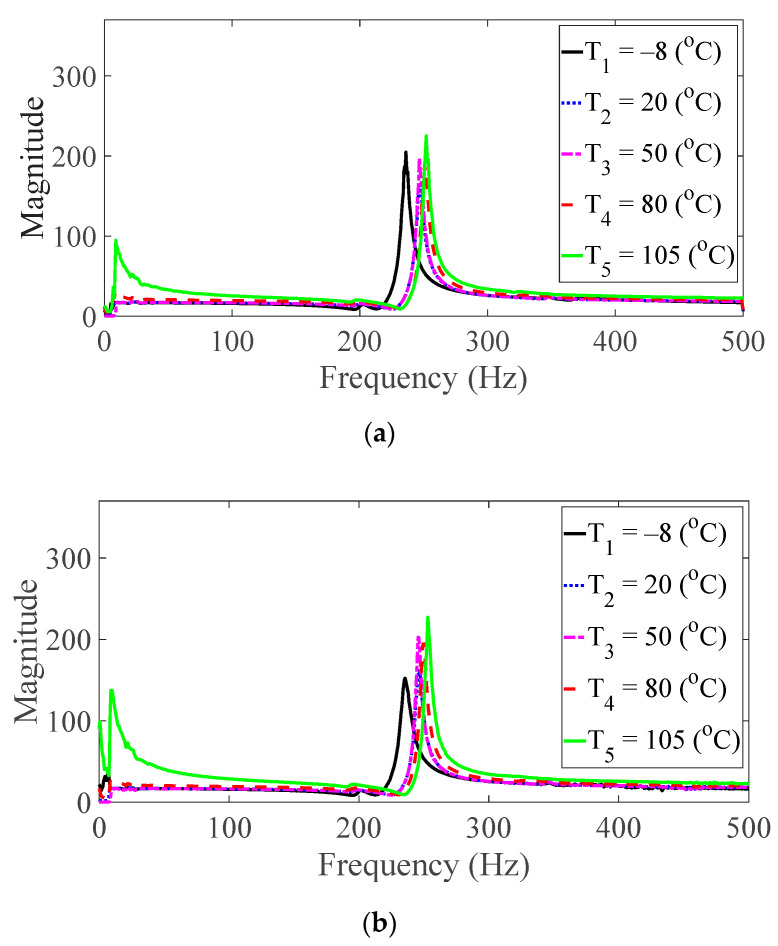
Measured FRFs with *θ* = 0°: (**a**) *p* = harmonic and (**b**) *p* = random.

**Figure 6:**
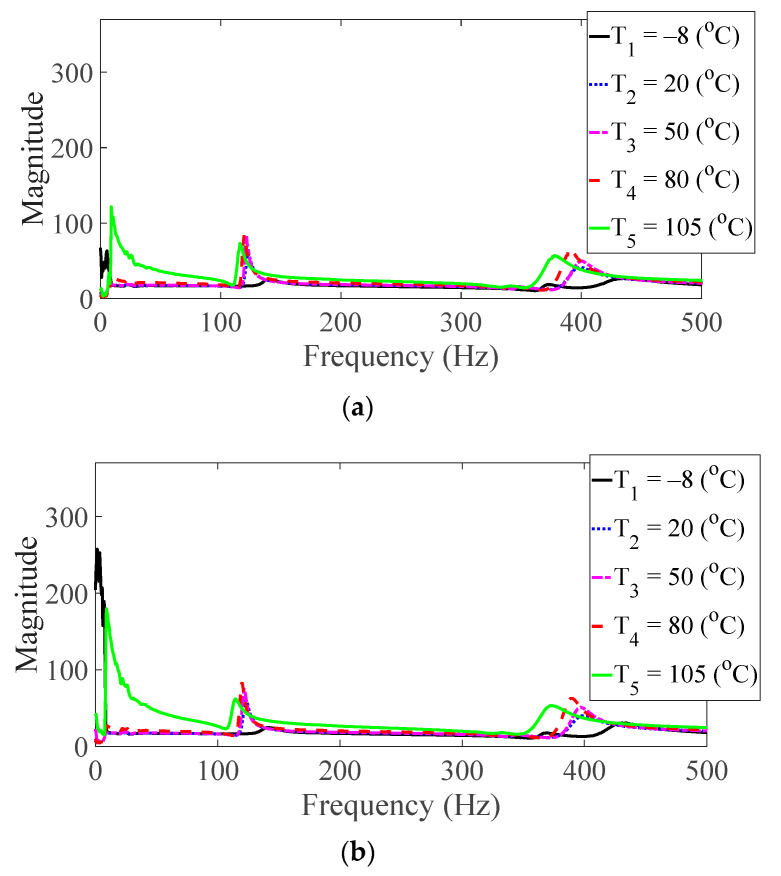
Measured FRFs with *θ* = 30°: (**a**) *p* = harmonic and (**b**) *p* = random.

**Figure 7:**
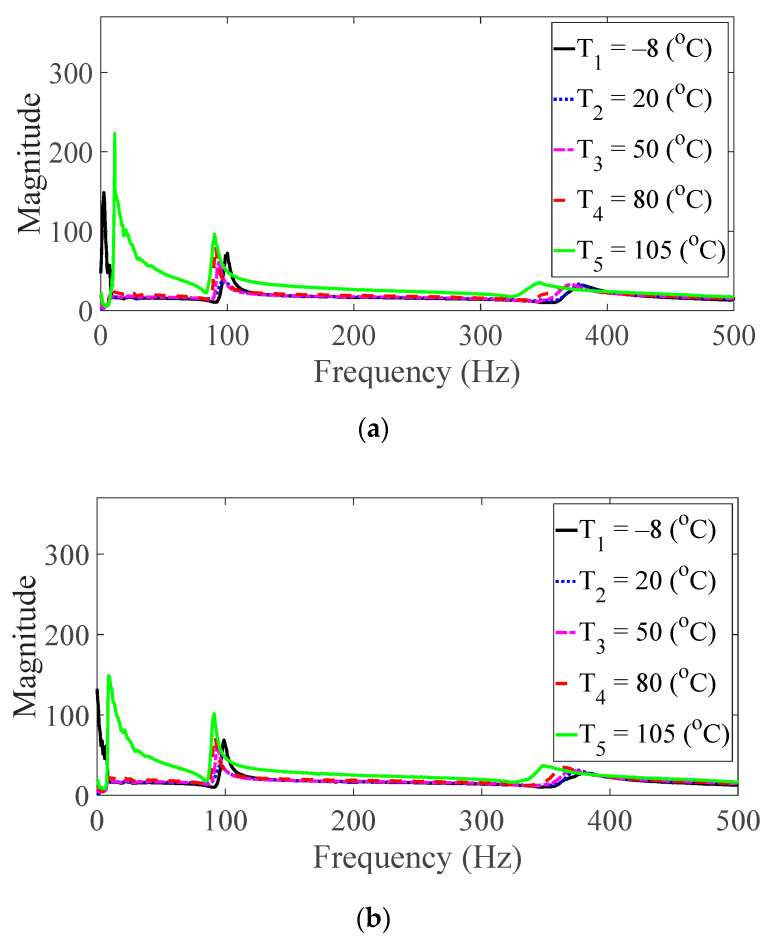
Measured FRFs with *θ* = 45°: (**a**) *p* = harmonic and (**b**) *p* = random.

**Figure 8:**
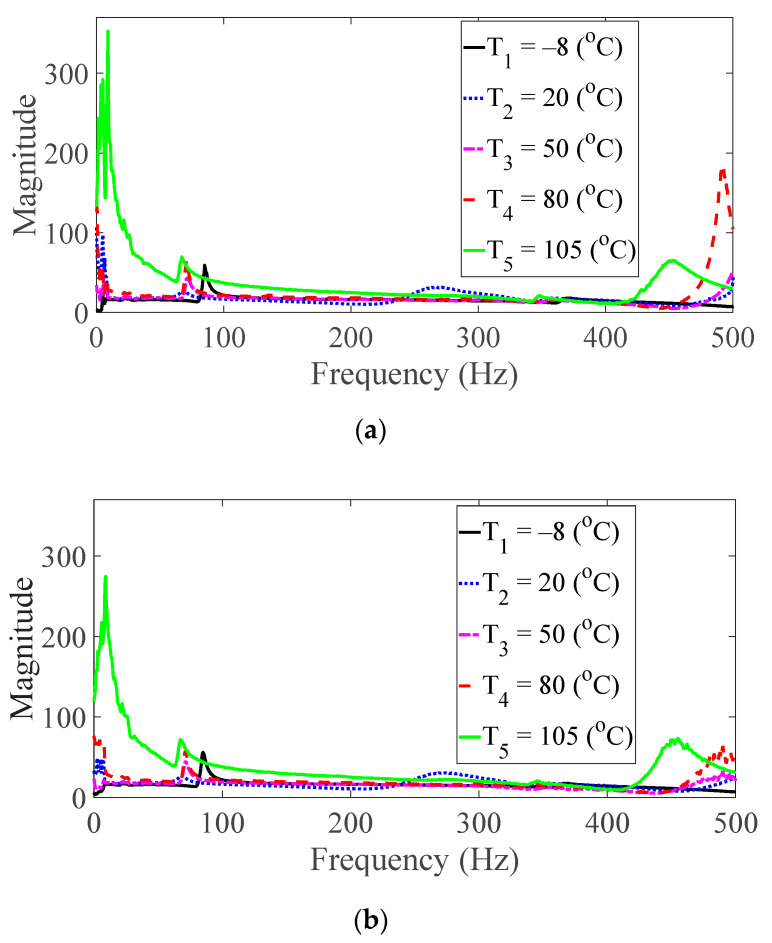
Measured FRFs with *θ* = 60°: (**a**) *p* = harmonic and (**b**) *p* = random.

**Figure 9:**
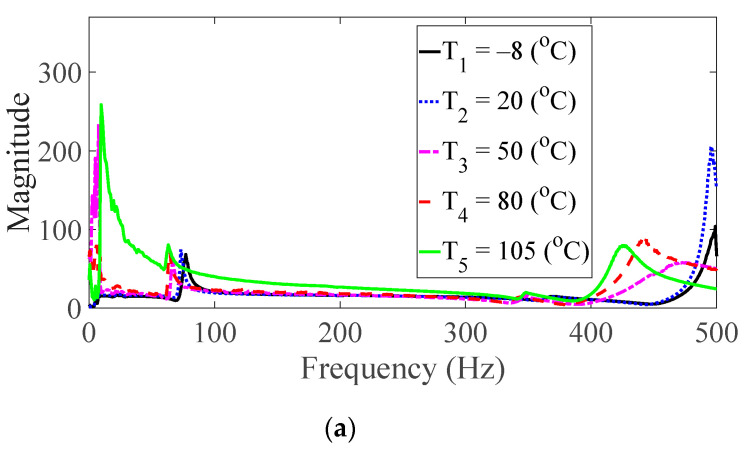

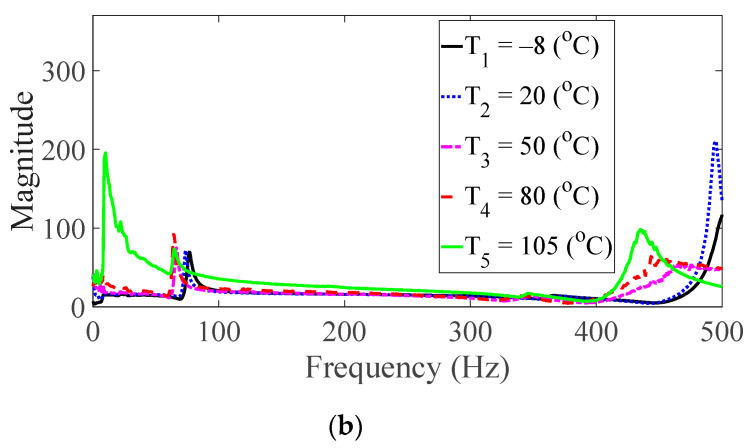
Measured FRFs with *θ* = 90°: (**a**) *p* = harmonic and (**b**) *p* = random.

Vibration tests were performed on five specimens using the vibration profiles listed in Table 1 and Table 2, and the force and acceleration data were simultaneously measured under five different temperature conditions. For the random spectral loading case, the FRFs were calculated via the average options, and for the harmonic excitation case, the FRF was calculated using the peak-hold options [25,26,27]. The measured FRF data were the same as those published in a previous study [24]. The FRFs were calculated by summing all seven frequencies under the same test condition, and the derived summation of the FRF was referred to as the representative FRF [24], as illustrated in Figure 5, Figure 6, Figure 7, Figure 8 and Figure 9. In addition, three CFRP specimens with the same direction of carbon fiber were tested and averaged under the same experimental conditions to minimize any truncation error during the experiments. The resonance frequency of interest was limited for the first resonance frequency, as the second resonance frequency exhibited different mode shapes according to the direction of the carbon fiber from the previous study [22]. The modal parameters, i.e., the resonance frequency and modal damping coefficient, were derived from the representative FRFs and are summarized in Table 4.

**Figure 10:**
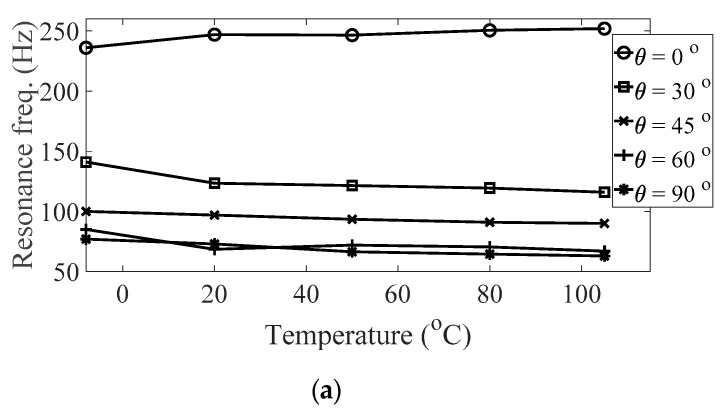

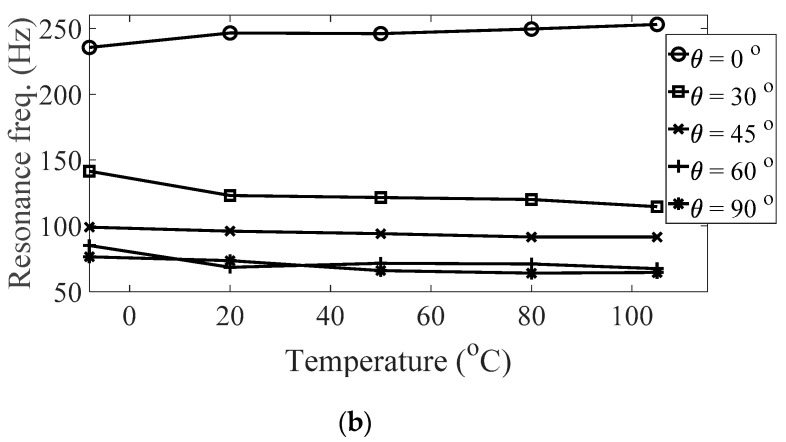
Variation of resonance frequency (*ω_n_*_,1_) according to different temperature conditions: (**a**) *p* = harmonic and (**b**) *p* = random.

**Figure 11:**
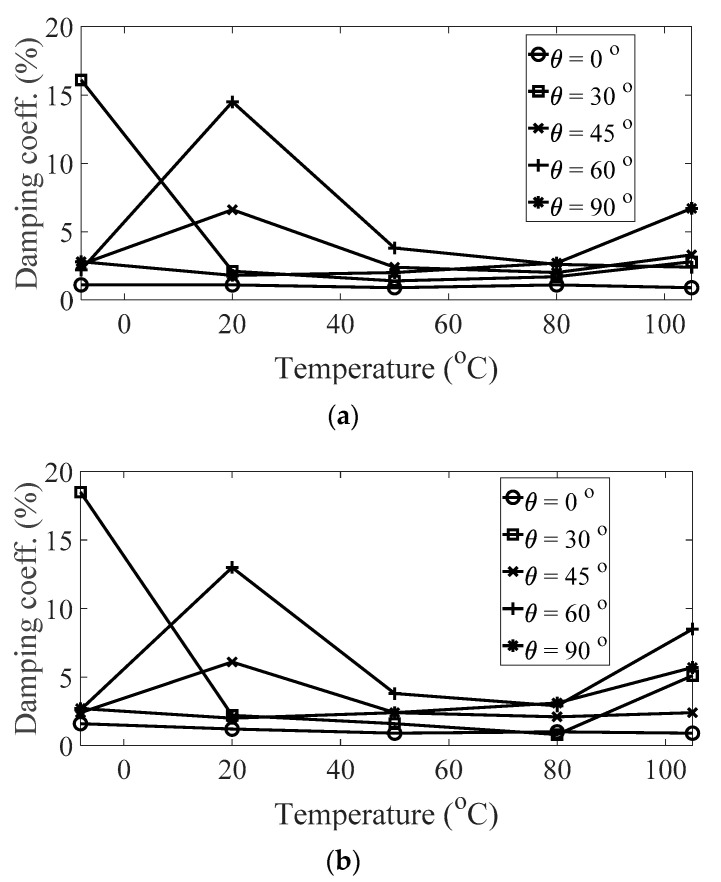
Variation of damping coefficient (*ξ*_1_) according to different temperature conditions: (**a**) *p* = harmonic and (**b**) *p* = random.

**Figure 12:**
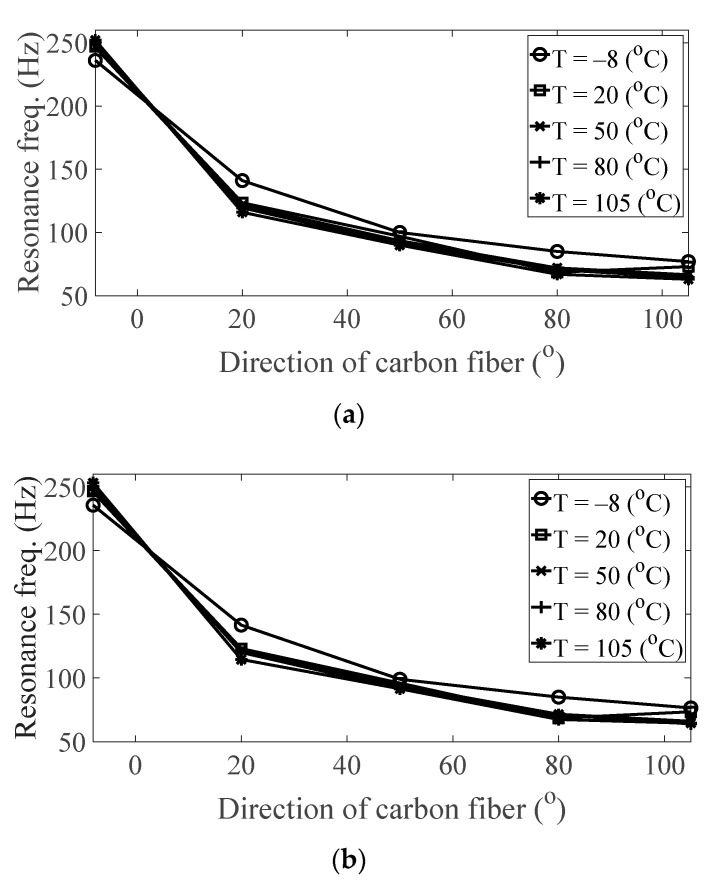
Variation of resonance frequency (*ω_n_*_,1_) according to different directions of carbon fiber: (**a**) *p* = harmonic and (**b**) *p* = random.

**Figure 13:**
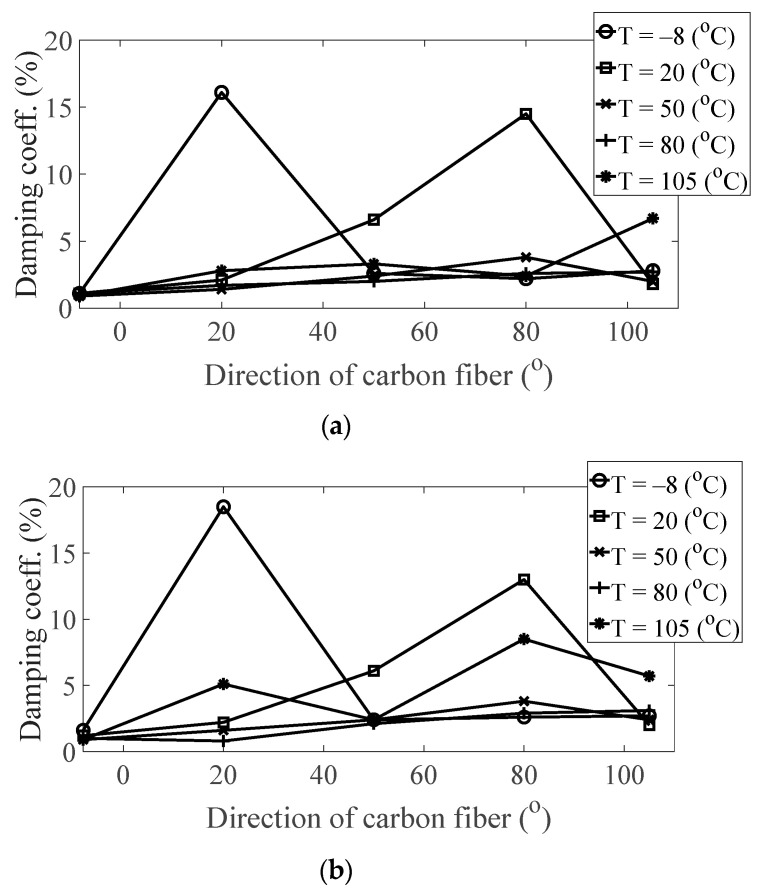
Variation of damping coefficient (*ξ*_1_) according to different directions of carbon fiber: (**a**) *p* = harmonic and (**b**) *p* = random.

The magnitude and peak frequency of the measured FRFs varied according to the different temperature conditions, as well as the direction of the carbon fiber except for the variation in the resonance frequency for different spectral loading patterns. The variation in the resonance frequency over the direction of the carbon fiber was reasonably acceptable due to the anisotropic nature of the carbon fiber. The dependency on temperature for the modal damping coefficient may be attributed to the time-dependent nature of the pre-implemented composite fibers because other parameters—both the direction of the carbon fiber and the spectral loading patterns—remained the same. However, further investigations into the pre-implemented composite fibers, including both the carbon fiber and the resin with respect to various temperature conditions, could not be conducted in this study because the related research required expertise in the field of chemical engineering. The variations in the FRF of the CFRP specimen were well-matched with the assumptions of the modal parameters in the theoretical FRF presented in Equation (5). In addition, changes in the modal parameters according to the three parameters of interest can be seen in Figure 10, Figure 11, Figure 12 and Figure 13. The variations in the modal parameters in each experimental case are further formulated as an approximated curve function in the following chapter.

## 4. Sensitivity Analysis

The variation in each parameter could be calculated via the curve-fitted function from the experimental results, as summarized in Table 4 and Table 5. Only the first resonance frequency of the CFRP specimen was considered in this study because the second mode shape in each specimen provided different modal analysis result values than those in a previous study [22]. The variation in the modal parameters, resonance frequency, and modal damping coefficient could be fitted for the two parameters of interest, i.e., the carbon fiber direction and temperature, and the curve-fitted third-order polynomial functions could be calculated using MATLAB Version: R2018b (MathWorks). The results are summarized in Table 6 and Table 7. The partial derivatives for the two parameters required in the normalized sensitivity index, i.e., temperature and carbon fiber direction, were calculated from the derivative of each approximated function. In addition, the partial derivatives for the spectral loading pattern were applied based on the relative error between the harmonic and random cases because the curve-fitting function was insufficient with just two cases, as presented in Table 8 [22]. The relative error between two different spectral loading patterns verified that the resonant frequency was independent of the spectral loading pattern parameter assumed in Equation (8).

**Figure 14:**
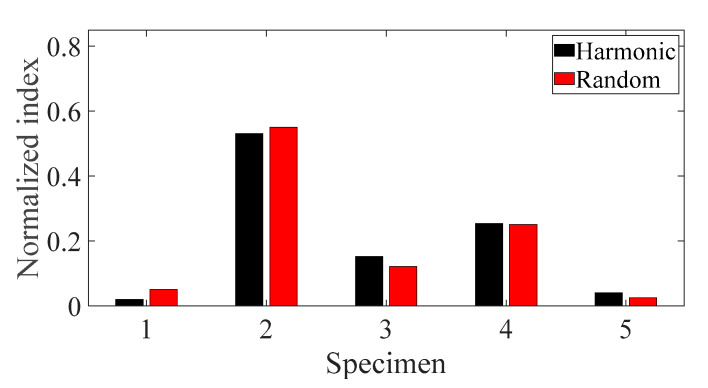
Normalized sensitivity index for the direction of carbon fiber for the two spectral loading cases.

**Figure 15:**
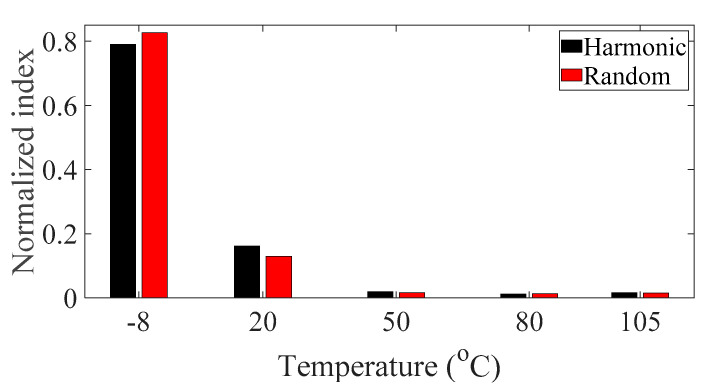
Normalized sensitivity index for the temperature for the two spectral loading cases.

**Figure 16:**
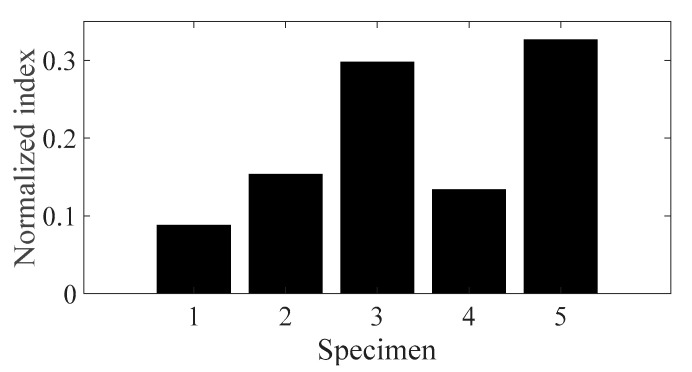
Normalized sensitivity indices for the spectral loading pattern.

The sensitivity analysis was conducted for five different specimens using the normalized sensitivity index in Equations (12)–(14), and the results are plotted in Figure 14, Figure 15 and Figure 16, respectively. Here, the *i*-number in the *x*-axis, in both Figure 14 and Figure 16, denotes the *i*th CFRP specimen with respect to the direction of the carbon fiber—*θ*_1_ = 0°, *θ*_2_ = 30°, *θ*_3_ = 45°, *θ*_4_ = 60°, and *θ*_5_ = 90°. In addition, the derivative of the modal damping coefficient between the two different spectral loading cases required in Equation (14) was calculated from the relative error between the two cases [22].

The normalized sensitivity index of specimen #1 was the smallest value for both parameters, i.e., temperature and spectral loading patterns; hence, the variation in the FRF of specimen #1 was more robust against the temperature condition and spectral loading pattern effect. For these two parameters, specimens #2 and #4 demonstrated higher values than the other specimens. During uniaxial excitation, the dynamic structural stiffness was also the highest in specimen #1; thus, it is best to design a CFRP specimen with a carbon fiber direction of 0° to deal with vertical loading. The effect of temperature on the CFRP specimen dynamics revealed that the normalized sensitivity value was the highest at −8 °C and lowest at 105 °C. Therefore, the variation in the CFRP specimen dynamics with carbon fiber direction was particularly sensitive in cold conditions and became less sensitive in hot conditions. In particular, the normalized sensitivity index result in this study did not match that in the previous study, although the same CFRP specimen was used for the uniaxial vibration test. In the previous study, the normalized sensitivity index was the highest for both parameters, i.e., the direction of the carbon fiber and the spectral loading pattern [22]. This discrepancy arose because the normalized sensitivity results in the present study were calculated considering all the temperature conditions from −8 to 105 °C and then summed for each specimen case; however, the sensitivity results in the previous study were only calculated for room temperature. Thus, the temperature condition has been demonstrated to be one of the most important parameters for considering CFRP structure dynamics.

## 5. Conclusions

The previous theoretical FRF of a CFRP specimen, which was dependent on two parameters (the direction of carbon fiber and spectral loading pattern) was extended to a three parameter-dependent FRF by including the temperature condition. The sensitivity indices of the FRF over the three parameters of interest were derived to evaluate the dynamics of the CFRP specimen with respect to each parameter case. The variation of modal parameters with the three parameters of interest was measured from a uniaxial excitation test, and the partial derivatives of the modal parameters were approximately calculated from curve-fitted polynomial functions. From previous studies [22,24], it was reasonably identified that the anisotropic nature of CFRP material changes its resonance frequency according to the direction of the carbon fiber; however, the variation in the modal damping parameters with the three parameters of interest is an important finding in the dynamic nature of CFRP materials that has not been included in previous studies. In particular, the variation in the modal damping coefficient with temperature may be related to chemical changes in the pre-implemented composite fibers, including both the carbon fiber and resin, and further investigations in this regard are left for future studies.

The sensitivity analysis results revealed that the dynamic characteristics of specimen #1 (*θ*_1_ = 0°) were the most robust over the two parameters, i.e., both the direction of the carbon fiber and the spectral loading pattern. Specimen #1 was found to have the highest resonance frequency; thus, the optimal carbon fiber direction is 0° for the uniaxial excitation situation. In addition, the sensitivity index demonstrated that the variation in dynamics for different carbon fiber directions was very high at the lowest temperature (−8 °C) and became low at the hottest temperature (105 °C). Therefore, CFRP structure dynamics are highly sensitive to the selection of carbon fiber at a low temperatures.

## Figures and Tables

**Table 1:** Test profile for random excitation.

No.	Frequency (Hz)	Acceleration (g^2^/Hz)
1	10	0.005
2	500	0.005

**Table 2:** Test profile for harmonic excitation.

No.	Frequency (Hz)	Acceleration (g)
1	10	0.5
2	500	0.5

**Table 3:** Temperature conditions in the uniaxial excitation test.

No.	Temperature
1	−8 °C
2	20 °C
3	50 °C
4	80 °C
5	105 °C

**Table 4:** Variation of the resonance frequency (*ω_n_*_,1_) according to different parameter conditions.

*θ*	Harmonic					Random				
	−8 °C	20 °C	50 °C	80 °C	105 °C	−8 °C	20 °C	50 °C	80 °C	105 °C
0°	230.6	247.0	246.5	250.5	252.0	235.5	246.5	246.0	249.5	253.0
30°	141.0	123.5	121.5	119.5	116.0	141.5	123.0	121.5	120.0	114.5
45°	100.0	97.0	93.5	91.0	90.0	99.0	96.0	94.0	91.5	91.5
60°	85.0	68.5	72.0	70.5	670	85.0	68.5	71.5	71.0	67.5
90°	77.0	73.0	73.0	64.5	63.0	76.5	73.5	66.0	64.0	64.5

**Table 5:** Variation of damping coefficient (*ξ*_1_) according to different parameter conditions.

*θ*	Harmonic					Random				
	−8 °C	20 °C	50 °C	80 °C	105 °C	−8 °C	20 °C	50 °C	80 °C	105 °C
0°	1.1	1.1	0.9	1.1	0.9	1.6	1.2	0.9	1.0	0.9
30°	16.1	2.1	1.4	1.7	2.8	18.5	2.2	1.6	0.8	5.1
45°	2.6	6.6	2.4	2.0	3.3	2.4	6.1	2.4	2.1	2.4
60°	2.2	14.5	3.8	2.6	2.4	2.6	13.0	3.8	2.9	8.5
90°	2.8	1.8	2.0	2.7	2.7	2.7	2.0	2.4	3.1	5.7

**Table 6:** Curve-fitted polynomial function of the first resonance frequency (*ω_n_*_,1_) with respect to the parameters of interest.

Parameter	Harmonic	Random
*θ*_1_ = 0°	−0.007·*T*^2^ + 0.40·*T* + 239.99	−0.008·*T*^2^ + 0.42·*T* + 239.66
*θ_2_* = 30°	−0.0001·*T*^3^ − 0.012·*T*^2^ − 0.71·*T* + 134.29	−0.0001·*T*^3^ − 0.014·*T*^2^ − 0.77·*T* + 134.20
*θ*_3_ = 45°	−0.0004·*T*^2^ − 0.111·*T* + 99.19	−0.0001·*T*^2^ − 0.095·*T* + 98.18
*θ*_4_ = 60°	−0.0001·*T*^3^ − 0.014·*T*^2^ − 0.66·*T* + 78.36	−0.0001·*T*^3^ + 0.014·*T*^2^ − 0.68·*T* + 78.28
*θ*_5_ = 90°	−0.0006·*T*^2^ − 0.17·*T* + 75.87	−0.0025·*T*^2^ − 0.11·*T* + 75.94
*T*_1_ = −8 °C	0.0001·*θ*^3^ + 0.018·*θ*^2^ − 3.86·*θ* + 236.29	0.0001·*θ*^3^ + 0.017·*θ*^2^ − 3.81·*θ* + 235.84
*T_2_* = 20 °C	−0.0001·*θ*^3^ + 0.041·*θ*^2^ − 5.19·*θ* + 246.66	−0.0001·*θ*^3^ + 0.042·*θ*^2^ − 5.21·*θ* + 246.18
*T_3_* = 50 °C	−0.0002·*θ*^3^ + 0.059·*θ*^2^ − 5.69·*θ* + 246.34	−0.0002·*θ*^3^ + 0.0597 − 5.63·*θ* + 245.81
*T_4_* = 80 °C	−0.0002·*θ*^3^ + 0.066·*θ*^2^ − 6.09·*θ* + 250.35	−0.0002·*θ*^3^ + 0.0665 − 6.02·*θ* + 249.36
*T_5_* = 105 °C	−0.0003·*θ*^3^ + 0.070·*θ*^2^ − 6.32·*θ* + 251.74	−0.0003·*θ*^3^ + 0.075·*θ*^2^ − 6.50·*θ* + 252.66

**Table 7:** Curve-fitted polynomial function of the first modal damping coefficient (ξ_1_) with respect to the parameters of interest.

Parameter	Harmonic	Random
*θ*_1_ = 0°	0.0001·*T*^2^ − 0.0046·*T* + 1.077	0.0002·*T*^2^ − 0.019·*T* + 1.44
*θ_2_* = 30°	0.0094·*T*^2^ − 0.57·*T* + 10.75	0.0092·*T*^2^ − 0.62·*T* + 12.57
*θ*_3_ = 45°	−0.0055·*T*^2^ − 0.16·*T* + 4.48	−0.0046·*T*^2^ − 0.15·*T* + 4.065
*θ*_4_ = 60°	0.0001·*T*^3^ − 0.014·*T*^2^ − 0.48·*T* + 7.60	0.0001·*T*^3^ − 0.014·*T*^2^ − 0.44·*T* + 7.53
*θ*_5_ = 90°	−0.0004·*T*^2^ − 0.022·*T* + 2.59	−0.019·*T* + 2.51
*T*_1_ = −8 °C	0.0003·*θ*^3^ − 0.039·*θ*^2^ + 1.32·*θ* + 1.38	0.0003·*θ*^3^ − 0.043·*θ*^2^ + 1.48·*θ* + 1.95
*T_2_* = 20 °C	−0.0002·*θ*^3^ + 0.027·*θ*^2^ − 0.61·*θ* + 1.20	−0.0002·*θ*^3^ + 0.024·*θ*^2^ − 0.52·*θ* + 1.29
*T_3_* = 50 °C	0.0045·*θ*^2^ − 0.089·*θ* + 0.91	0.0038·*θ*^2^ − 0.067·*θ* + 0.92
*T_4_* = 80 °C	0.0008·*θ*^2^ − 0.0007·*θ* + 1.11	0.0036·*θ*^2^ − 0.086·*θ* + 0.99
*T_5_* = 105 °C	−0.0053·*θ*^2^ + 0.20·*θ* + 0.87	−0.0036·*θ*^2^ + 0.0012·*θ* + 1.084

**Table 8:** Relative error in the first modal parameters (unit: %).

*θ*	Resonance Frequency					Modal Damping Coefficient				
	−8 °C	20 °C	50 °C	80 °C	105 °C	−8 °C	20 °C	50 °C	80 °C	105 °C
0°	0.21	0.20	0.20	0.40	0.40	45.45	9.09	0.0	9.09	0.0
30°	0.35	0.40	0.0	0.42	1.29	14.91	4.76	14.29	52.94	82.14
45°	1.00	1.03	0.53	0.55	1.67	7.69	7.58	0.0	5.00	27.27
60°	0.0	0.0	0.69	0.71	0.75	18.18	10.34	0.0	11.54	254.17
90°	0.65	0.68	0.75	0.78	2.38	3.57	11.11	20.00	14.81	14.93
